# (3,5,7-Tribromo­tropolonato-κ^2^ *O*,*O*′)tris­(triphenyl­phosphine-κ*P*)silver(I)

**DOI:** 10.1107/S1600536809000890

**Published:** 2009-01-14

**Authors:** G. Steyl, T. N. Hill

**Affiliations:** a Department of Chemistry, University of the Free State, Bloemfontein 9300, South Africa

## Abstract

The title compound, [Ag(C_7_H_2_Br_3_O_2_)(C_18_H_15_P)_3_], a silver(I) derivative of 3,5,7-tribromo­tropolone, has three triphenyl­phosphine ligands coordinated to the silver centre, whereas the 3,5,7-tribromo­tropolonate anion coordinates as a bidentate ligand. The compound is an example of a five-coordinate silver complex containing a bidentate ligand.

## Related literature

The title compound is structurally related to other silver tropolonato and oxalato derivatives; see: Steyl & Hill (2009 ▶); Dean *et al.* (2001 ▶). For diketonato complexes, see: Hill & Steyl (2008 ▶); Steyl (2006 ▶, 2007 ▶); Steyl & Hill (2009 ▶); Steyl & Roodt (2006 ▶). For general background, see: Roodt *et al.* (2003 ▶); Crous *et al.* (2005 ▶).
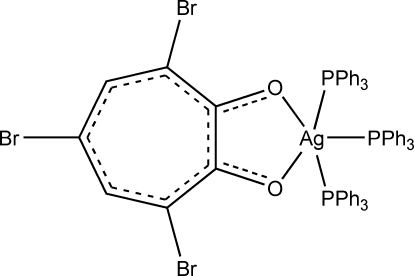


## Experimental

### 

#### Crystal data

[Ag(C_7_H_2_Br_3_O_2_)(C_18_H_15_P)_3_]
*M* *_r_* = 1252.50
Monoclinic, 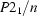

*a* = 13.257 (1) Å
*b* = 27.1550 (17) Å
*c* = 14.688 (1) Å
β = 93.121 (2)°
*V* = 5279.7 (6) Å^3^
*Z* = 4
Mo *K*α radiation
μ = 2.79 mm^−1^
*T* = 100 (2) K
0.29 × 0.15 × 0.09 mm

#### Data collection

Bruker APEXII area-detector diffractometer
Absorption correction: multi-scan (*SADABS*; Bruker, 1998 ▶) *T*_min_ = 0.499, *T*_max_ = 0.788
74600 measured reflections
11519 independent reflections
7888 reflections with *I* > 2σ(*I*)
*R*_int_ = 0.107

#### Refinement

*R*[*F* ^2^ > 2σ(*F* ^2^)] = 0.046
*wR*(*F* ^2^) = 0.165
*S* = 1.07
11519 reflections
631 parameters
H-atom parameters constrained
Δρ_max_ = 0.72 e Å^−3^
Δρ_min_ = −1.18 e Å^−3^

### 

Data collection: *APEX2* (Bruker, 2005 ▶); cell refinement: *SAINT-Plus* (Bruker, 2004 ▶); data reduction: *SAINT-Plus*; program(s) used to solve structure: *SHELXS97* (Sheldrick, 2008 ▶); program(s) used to refine structure: *SHELXL97* (Sheldrick, 2008 ▶); molecular graphics: *DIAMOND* (Brandenburg & Putz, 2006 ▶); software used to prepare material for publication: *SHELXL97*.

## Supplementary Material

Crystal structure: contains datablocks I, global. DOI: 10.1107/S1600536809000890/ng2535sup1.cif

Structure factors: contains datablocks I. DOI: 10.1107/S1600536809000890/ng2535Isup2.hkl

Additional supplementary materials:  crystallographic information; 3D view; checkCIF report

## Figures and Tables

**Table:** 

Ag—O1	2.630 (4)
Ag—O2	2.597 (4)
Ag—P1	2.5412 (14)
Ag—P2	2.5198 (14)
Ag—P3	2.5160 (14)

**Table:** 

O2—Ag—O1	59.36 (12)
P3—Ag—P2	112.98 (5)
P3—Ag—P1	112.59 (5)
P2—Ag—P1	118.42 (5)
P3—Ag—O2	105.72 (10)
P2—Ag—O2	126.43 (10)
P1—Ag—O2	75.45 (9)
P3—Ag—O1	81.79 (9)
P2—Ag—O1	90.69 (9)
P1—Ag—O1	134.81 (9)

**Table:** 

O1—C1—C2—O2	−6.1 (7)

**Fig. 1.**
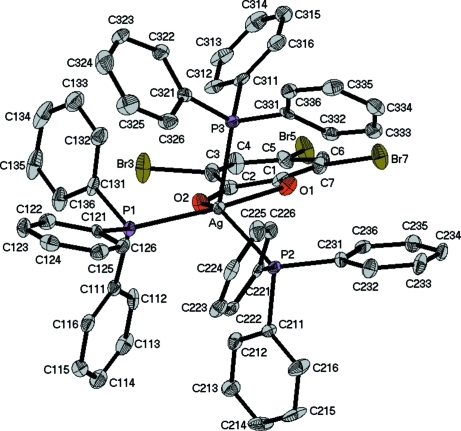
: Representation of the title compound (I), showing the numbering scheme and displacement ellipsoids (50% probability). For the carbon rings on the triphenylphosphine ligands, the first digit refers to phosphorous number, second digit to the ring number and third digit to atom in the ring. Hydrogen atoms omitted for clarity.

## supplementary crystallographic information

### Comment

Recently, solid state studies containing the 3,5,7-tribromotropolone ligand have been reported (Steyl, 2007; Steyl & Roodt, 2006; Roodt *et al.*, 2003; Crous *et al.*, 2005), different metal centers such as Rh(I) and Pd(I) have been investigated. In this regard, we present a silver(I) tris(triphenylphosphine) complex of the 3,5,7-tribromotropolonato moiety.

The Ag—O bond distances are closely related in length while the Ag—P bond distances differ from each other, Table 1. A single phosphorous atom (P1) has a slight increase in bond distance compared to P2 and P3. Comparing the title compound to a similar tropolonato complex (Steyl & Hill, 2009) the Ag—O bond distances increases by *ca* 0.2 Å, while the Ag—P distances increased by *ca* 0.1 Å. This trend is indicative of the higher coordination number about the silver metal centre and the weaker coordination properties of the 3,5,7-tribromotropolonato moiety. The bidentate bite angle of the title compound is nearly 10 ° smaller than the related tropolonato or oxolato complex (Steyl & Hill, 2009; Dean *et al.*, 2001).

In previous studies the longest Ag—O bond distance reported for the 3,5,7-tribromotropolonato moiety was *ca* 2.496 Å (Roodt *et al.*, 2003), while in the title compound this bond length has increased to 2.630 (4) Å. The increase in coordination number of the silver moiety is a direct result of the weaker coordinating ability of the bidentate ligand compared to the related tropolonato complex, which contains only two triphenylphosphine ligands.

### Experimental

The title complex was synthesized by the addition of the sodium salt of 3,5,7-tribromotropolone (204 mg, 0.57 mmol) to an dichloromethane solution (10 ml) of the [Cu(PPh_3_)_2_NO_3_] (370 mg, 0.57 mmol). On slow evaporation of the solvent; crystals suitable for X-Ray crystallography was obtained. Yield: 400 mg (58%).

### Refinement

H atoms were positioned geometrically and refined using a riding model, with C—H = 0.95 Å and *U*_iso_(H) = 1.2 times *U*_eq_(C aromatic).

### Crystal data

**Table:**

[Ag(C_7_H_2_Br_3_O_2_)(C_18_H_15_P)_3_]	*F*(000) = 2504
*M**_r_* = 1252.50	*D*_x_ = 1.576 Mg m^−^^3^
Monoclinic, *P*2_1_/*n*	Mo *K*α radiation, λ = 0.71073 Å
Hall symbol: -P 2yn	Cell parameters from 5934 reflections
*a* = 13.257 (1) Å	θ = 3.1–22.7°
*b* = 27.1550 (17) Å	µ = 2.79 mm^−^^1^
*c* = 14.688 (1) Å	*T* = 100 K
β = 93.121 (2)°	Cuboid, yellow
*V* = 5279.7 (6) Å^3^	0.29 × 0.15 × 0.09 mm
*Z* = 4	

### Data collection

**Table:**

Bruker 4K CCD area-detector diffractometer	11519 independent reflections
Radiation source: fine-focus sealed tube	7888 reflections with *I* > 2σ(*I*)
graphite	*R*_int_ = 0.107
Detector resolution: 512 x 512 pixels mm^-1^	θ_max_ = 27.0°, θ_min_ = 1.6°
φ and ω scans	*h* = −16→16
Absorption correction: multi-scan (*SADABS*; Bruker,1998)	*k* = −34→34
*T*_min_ = 0.499, *T*_max_ = 0.788	*l* = −18→18
74600 measured reflections	

### Refinement

**Table:**

Refinement on *F*^2^	Primary atom site location: structure-invariant direct methods
Least-squares matrix: full	Secondary atom site location: difference Fourier map
*R*[*F*^2^ > 2σ(*F*^2^)] = 0.046	Hydrogen site location: riding model
*wR*(*F*^2^) = 0.165	H-atom parameters constrained
*S* = 1.07	*w* = 1/[σ^2^(*F*_o_^2^) + (0.087*P*)^2^ + 1.428*P*] where *P* = (*F*_o_^2^ + 2*F*_c_^2^)/3
11519 reflections	(Δ/σ)_max_ = 0.002
631 parameters	Δρ_max_ = 0.72 e Å^−^^3^
0 restraints	Δρ_min_ = −1.18 e Å^−^^3^

### Special details

**Table:**

Geometry. All e.s.d.'s (except the e.s.d. in the dihedral angle between two l.s. planes) are estimated using the full covariance matrix. The cell e.s.d.'s are taken into account individually in the estimation of e.s.d.'s in distances, angles and torsion angles; correlations between e.s.d.'s in cell parameters are only used when they are defined by crystal symmetry. An approximate (isotropic) treatment of cell e.s.d.'s is used for estimating e.s.d.'s involving l.s. planes.
Refinement. Refinement of *F*^2^ against ALL reflections. The weighted *R*-factor *wR* and goodness of fit *S* are based on *F*^2^, conventional *R*-factors *R* are based on *F*, with *F* set to zero for negative *F*^2^. The threshold expression of *F*^2^ > σ(*F*^2^) is used only for calculating *R*-factors(gt) *etc*. and is not relevant to the choice of reflections for refinement. *R*-factors based on *F*^2^ are statistically about twice as large as those based on *F*, and *R*- factors based on ALL data will be even larger.

### Fractional atomic coordinates and isotropic or equivalent isotropic displacement parameters (Å^2^)

**Table:**

	*x*	*y*	*z*	*U*_iso_*/*U*_eq_	
Ag	0.18844 (3)	0.125664 (14)	0.68777 (3)	0.01393 (12)	
Br7	0.20169 (5)	−0.04255 (2)	0.89448 (5)	0.03177 (18)	
Br5	0.51227 (5)	−0.13951 (2)	0.75005 (5)	0.03368 (18)	
Br3	0.53351 (5)	0.04342 (2)	0.59589 (5)	0.0392 (2)	
P2	0.00007 (10)	0.11235 (5)	0.67588 (9)	0.0134 (3)	
P1	0.27008 (10)	0.16932 (5)	0.55760 (10)	0.0149 (3)	
P3	0.25150 (10)	0.15700 (5)	0.84172 (10)	0.0154 (3)	
C1	0.2851 (4)	0.0111 (2)	0.7538 (4)	0.0188 (12)	
O1	0.2149 (3)	0.04018 (13)	0.7698 (3)	0.0217 (9)	
O2	0.3253 (3)	0.06463 (14)	0.6370 (3)	0.0269 (10)	
C331	0.1569 (4)	0.15493 (19)	0.9278 (3)	0.0162 (12)	
C335	0.0551 (4)	0.1884 (2)	1.0431 (4)	0.0257 (14)	
H335	0.0375	0.2154	1.0802	0.031*	
C221	−0.0631 (4)	0.1724 (2)	0.6815 (4)	0.0170 (12)	
C121	0.2327 (4)	0.23395 (19)	0.5429 (4)	0.0151 (11)	
C111	0.2443 (4)	0.1441 (2)	0.4426 (4)	0.0171 (12)	
C211	−0.0487 (4)	0.08484 (19)	0.5679 (4)	0.0154 (11)	
C132	0.4522 (4)	0.1880 (2)	0.6530 (4)	0.0265 (14)	
H132	0.4103	0.1970	0.7007	0.032*	
C234	−0.1611 (4)	0.0118 (2)	0.8766 (4)	0.0235 (13)	
H234	−0.1935	−0.0100	0.9163	0.028*	
C333	0.0318 (5)	0.1043 (2)	0.9974 (4)	0.0257 (14)	
H333	−0.0021	0.0736	1.0021	0.031*	
C223	−0.1490 (4)	0.2413 (2)	0.6089 (4)	0.0241 (14)	
H223	−0.1775	0.2568	0.5555	0.029*	
C116	0.1990 (4)	0.1717 (2)	0.3717 (4)	0.0209 (13)	
H116	0.1844	0.2056	0.3809	0.025*	
C2	0.3516 (4)	0.0270 (2)	0.6797 (4)	0.0189 (12)	
C225	−0.1072 (5)	0.2430 (2)	0.7696 (4)	0.0274 (14)	
H225	−0.1086	0.2595	0.8266	0.033*	
C332	0.1061 (4)	0.1095 (2)	0.9360 (4)	0.0191 (12)	
H332	0.1233	0.0825	0.8989	0.023*	
C122	0.2955 (4)	0.2692 (2)	0.5055 (4)	0.0206 (12)	
H122	0.3623	0.2605	0.4915	0.025*	
C224	−0.1507 (4)	0.2647 (2)	0.6918 (4)	0.0258 (14)	
H224	−0.1821	0.2961	0.6957	0.031*	
C321	0.2960 (4)	0.2205 (2)	0.8439 (4)	0.0183 (12)	
C113	0.2411 (5)	0.0736 (2)	0.3436 (4)	0.0275 (15)	
H113	0.2565	0.0399	0.3336	0.033*	
C212	0.0186 (4)	0.07450 (19)	0.5013 (4)	0.0200 (12)	
H212	0.0882	0.0821	0.5116	0.024*	
C311	0.3563 (4)	0.12088 (19)	0.8937 (4)	0.0199 (12)	
C4	0.4843 (5)	−0.0432 (2)	0.6857 (4)	0.0259 (14)	
H4	0.5454	−0.0522	0.6593	0.031*	
C322	0.3932 (4)	0.2336 (2)	0.8765 (4)	0.0229 (13)	
H322	0.4371	0.2096	0.9041	0.027*	
C131	0.4080 (4)	0.1730 (2)	0.5686 (4)	0.0201 (13)	
C222	−0.1059 (4)	0.1953 (2)	0.6030 (4)	0.0190 (12)	
H222	−0.1051	0.1791	0.5457	0.023*	
C336	0.1288 (4)	0.1942 (2)	0.9810 (4)	0.0202 (12)	
H336	0.1604	0.2253	0.9747	0.024*	
C5	0.4426 (4)	−0.0778 (2)	0.7413 (4)	0.0210 (13)	
C334	0.0063 (4)	0.1432 (2)	1.0518 (4)	0.0256 (14)	
H334	−0.0440	0.1393	1.0948	0.031*	
C124	0.1642 (4)	0.3295 (2)	0.5106 (4)	0.0228 (13)	
H124	0.1404	0.3621	0.4990	0.027*	
C236	−0.0213 (4)	0.0302 (2)	0.7841 (4)	0.0175 (12)	
H236	0.0419	0.0213	0.7614	0.021*	
C215	−0.1836 (5)	0.0513 (2)	0.4706 (5)	0.0342 (16)	
H215	−0.2529	0.0433	0.4593	0.041*	
C325	0.2657 (5)	0.3049 (2)	0.7960 (5)	0.0361 (17)	
H325	0.2221	0.3292	0.7691	0.043*	
C6	0.3602 (4)	−0.0740 (2)	0.7951 (4)	0.0186 (12)	
H6	0.3456	−0.1022	0.8303	0.022*	
C112	0.2642 (4)	0.0942 (2)	0.4274 (4)	0.0222 (13)	
H112	0.2938	0.0745	0.4753	0.027*	
C123	0.2613 (4)	0.3164 (2)	0.4890 (4)	0.0232 (13)	
H123	0.3040	0.3400	0.4629	0.028*	
C213	−0.0155 (5)	0.0532 (2)	0.4202 (4)	0.0266 (14)	
H213	0.0303	0.0467	0.3742	0.032*	
C231	−0.0663 (4)	0.07515 (19)	0.7595 (4)	0.0167 (12)	
C313	0.5055 (5)	0.0732 (2)	0.8722 (5)	0.0304 (15)	
H313	0.5552	0.0615	0.8331	0.037*	
C126	0.1365 (4)	0.2476 (2)	0.5652 (4)	0.0191 (12)	
H126	0.0938	0.2241	0.5918	0.023*	
C312	0.4283 (4)	0.1029 (2)	0.8365 (4)	0.0234 (13)	
H312	0.4244	0.1110	0.7735	0.028*	
C115	0.1753 (4)	0.1501 (2)	0.2878 (4)	0.0253 (14)	
H115	0.1445	0.1692	0.2397	0.030*	
C326	0.2324 (5)	0.2562 (2)	0.8032 (4)	0.0254 (14)	
H326	0.1664	0.2474	0.7803	0.031*	
C7	0.2968 (4)	−0.0340 (2)	0.8035 (4)	0.0195 (12)	
C315	0.4415 (5)	0.0779 (2)	1.0187 (5)	0.0332 (16)	
H315	0.4463	0.0692	1.0814	0.040*	
C235	−0.0696 (4)	−0.0012 (2)	0.8416 (4)	0.0222 (13)	
H235	−0.0398	−0.0321	0.8574	0.027*	
C233	−0.2047 (5)	0.0565 (2)	0.8533 (4)	0.0260 (14)	
H233	−0.2667	0.0658	0.8780	0.031*	
C133	0.5555 (5)	0.1899 (2)	0.6675 (5)	0.0356 (17)	
H133	0.5845	0.1999	0.7252	0.043*	
C125	0.1017 (4)	0.2951 (2)	0.5492 (4)	0.0229 (13)	
H125	0.0355	0.3041	0.5646	0.027*	
C216	−0.1505 (4)	0.0728 (2)	0.5525 (4)	0.0291 (15)	
H216	−0.1970	0.0794	0.5979	0.035*	
C226	−0.0618 (4)	0.19746 (19)	0.7645 (4)	0.0217 (13)	
H226	−0.0296	0.1831	0.8175	0.026*	
C324	0.3626 (5)	0.3176 (2)	0.8282 (5)	0.0355 (16)	
H324	0.3855	0.3505	0.8230	0.043*	
C114	0.1960 (5)	0.1010 (2)	0.2735 (4)	0.0302 (15)	
H114	0.1794	0.0862	0.2160	0.036*	
C214	−0.1154 (5)	0.0415 (2)	0.4064 (4)	0.0317 (16)	
H214	−0.1381	0.0262	0.3508	0.038*	
C135	0.5754 (5)	0.1639 (2)	0.5133 (6)	0.0391 (18)	
H135	0.6182	0.1560	0.4655	0.047*	
C316	0.3634 (5)	0.1084 (2)	0.9857 (4)	0.0270 (14)	
H316	0.3153	0.1207	1.0257	0.032*	
C134	0.6166 (5)	0.1774 (2)	0.5989 (6)	0.0412 (19)	
H134	0.6878	0.1779	0.6098	0.049*	
C3	0.4466 (4)	0.0034 (2)	0.6641 (4)	0.0224 (13)	
C136	0.4705 (5)	0.1620 (2)	0.4984 (5)	0.0286 (15)	
H136	0.4418	0.1531	0.4400	0.034*	
C314	0.5119 (5)	0.0602 (2)	0.9614 (5)	0.0360 (17)	
H314	0.5646	0.0392	0.9843	0.043*	
C323	0.4253 (5)	0.2824 (2)	0.8678 (4)	0.0298 (15)	
H323	0.4915	0.2913	0.8897	0.036*	
C232	−0.1584 (4)	0.0880 (2)	0.7941 (4)	0.0223 (13)	
H232	−0.1895	0.1184	0.7772	0.027*	

### Atomic displacement parameters (Å^2^)

**Table:**

	*U*^11^	*U*^22^	*U*^33^	*U*^12^	*U*^13^	*U*^23^
Ag	0.0115 (2)	0.0155 (2)	0.0148 (2)	−0.00044 (15)	0.00092 (15)	0.00050 (17)
Br7	0.0295 (4)	0.0335 (4)	0.0341 (4)	0.0099 (3)	0.0177 (3)	0.0095 (3)
Br5	0.0424 (4)	0.0241 (3)	0.0364 (4)	0.0178 (3)	0.0186 (3)	0.0099 (3)
Br3	0.0361 (4)	0.0277 (4)	0.0563 (5)	0.0034 (3)	0.0237 (3)	0.0152 (3)
P2	0.0098 (6)	0.0158 (7)	0.0146 (7)	−0.0010 (5)	0.0009 (5)	−0.0021 (6)
P1	0.0127 (7)	0.0154 (7)	0.0168 (7)	−0.0009 (5)	0.0032 (6)	0.0014 (6)
P3	0.0143 (7)	0.0156 (7)	0.0159 (7)	−0.0021 (6)	−0.0027 (6)	0.0009 (6)
C1	0.013 (3)	0.016 (3)	0.027 (3)	−0.003 (2)	−0.004 (2)	−0.002 (2)
O1	0.014 (2)	0.017 (2)	0.035 (2)	0.0029 (16)	0.0026 (17)	−0.0031 (18)
O2	0.031 (2)	0.019 (2)	0.031 (2)	0.0087 (18)	0.0027 (19)	0.0093 (19)
C331	0.021 (3)	0.017 (3)	0.010 (3)	0.002 (2)	−0.006 (2)	0.004 (2)
C335	0.022 (3)	0.024 (3)	0.030 (3)	0.004 (3)	−0.005 (3)	−0.005 (3)
C221	0.008 (3)	0.019 (3)	0.025 (3)	−0.006 (2)	0.003 (2)	0.002 (2)
C121	0.014 (3)	0.017 (3)	0.013 (3)	0.000 (2)	−0.002 (2)	0.008 (2)
C111	0.017 (3)	0.019 (3)	0.016 (3)	−0.003 (2)	0.006 (2)	−0.002 (2)
C211	0.013 (3)	0.017 (3)	0.016 (3)	−0.001 (2)	0.000 (2)	−0.003 (2)
C132	0.019 (3)	0.026 (3)	0.034 (4)	−0.002 (3)	−0.003 (3)	0.008 (3)
C234	0.028 (3)	0.022 (3)	0.021 (3)	−0.014 (3)	0.007 (3)	−0.002 (3)
C333	0.030 (3)	0.023 (3)	0.023 (3)	−0.005 (3)	−0.005 (3)	0.004 (3)
C223	0.013 (3)	0.020 (3)	0.039 (4)	0.001 (2)	−0.005 (3)	0.004 (3)
C116	0.023 (3)	0.016 (3)	0.024 (3)	−0.007 (2)	0.006 (2)	−0.002 (2)
C2	0.022 (3)	0.017 (3)	0.018 (3)	0.002 (2)	0.001 (2)	−0.008 (2)
C225	0.039 (4)	0.017 (3)	0.028 (4)	−0.006 (3)	0.009 (3)	−0.003 (3)
C332	0.026 (3)	0.015 (3)	0.016 (3)	0.000 (2)	−0.005 (2)	−0.001 (2)
C122	0.015 (3)	0.023 (3)	0.024 (3)	0.002 (2)	0.000 (2)	−0.001 (3)
C224	0.019 (3)	0.019 (3)	0.040 (4)	−0.006 (2)	0.007 (3)	0.000 (3)
C321	0.022 (3)	0.017 (3)	0.016 (3)	−0.003 (2)	−0.002 (2)	0.001 (2)
C113	0.026 (3)	0.021 (3)	0.037 (4)	−0.006 (3)	0.016 (3)	−0.014 (3)
C212	0.021 (3)	0.016 (3)	0.024 (3)	−0.003 (2)	0.005 (2)	−0.002 (2)
C311	0.017 (3)	0.013 (3)	0.029 (3)	−0.006 (2)	−0.010 (2)	0.003 (2)
C4	0.025 (3)	0.021 (3)	0.032 (4)	0.007 (2)	0.009 (3)	0.002 (3)
C322	0.026 (3)	0.019 (3)	0.023 (3)	−0.005 (2)	−0.004 (3)	0.002 (3)
C131	0.011 (3)	0.015 (3)	0.034 (3)	0.000 (2)	0.004 (2)	0.009 (3)
C222	0.012 (3)	0.022 (3)	0.023 (3)	−0.005 (2)	0.000 (2)	−0.002 (3)
C336	0.020 (3)	0.020 (3)	0.020 (3)	−0.002 (2)	−0.001 (2)	−0.001 (2)
C5	0.028 (3)	0.016 (3)	0.019 (3)	0.007 (2)	0.002 (2)	0.004 (2)
C334	0.018 (3)	0.040 (4)	0.019 (3)	0.006 (3)	0.002 (2)	0.004 (3)
C124	0.031 (3)	0.015 (3)	0.022 (3)	0.006 (2)	−0.004 (3)	−0.001 (2)
C236	0.013 (3)	0.021 (3)	0.018 (3)	0.000 (2)	−0.002 (2)	−0.002 (2)
C215	0.016 (3)	0.044 (4)	0.041 (4)	−0.007 (3)	−0.009 (3)	−0.016 (3)
C325	0.040 (4)	0.020 (3)	0.046 (4)	0.002 (3)	−0.013 (3)	0.007 (3)
C6	0.020 (3)	0.019 (3)	0.018 (3)	−0.003 (2)	0.004 (2)	0.002 (2)
C112	0.026 (3)	0.014 (3)	0.027 (3)	−0.002 (2)	0.012 (3)	0.004 (3)
C123	0.021 (3)	0.020 (3)	0.029 (3)	−0.002 (2)	0.002 (3)	0.010 (3)
C213	0.025 (3)	0.035 (4)	0.021 (3)	−0.006 (3)	0.005 (3)	−0.010 (3)
C231	0.016 (3)	0.019 (3)	0.016 (3)	−0.002 (2)	0.003 (2)	0.002 (2)
C313	0.021 (3)	0.021 (3)	0.048 (4)	0.000 (3)	−0.010 (3)	−0.007 (3)
C126	0.015 (3)	0.020 (3)	0.022 (3)	−0.004 (2)	0.002 (2)	0.000 (2)
C312	0.018 (3)	0.022 (3)	0.029 (3)	−0.001 (2)	−0.008 (3)	−0.004 (3)
C115	0.027 (3)	0.029 (3)	0.020 (3)	−0.007 (3)	0.001 (3)	0.001 (3)
C326	0.027 (3)	0.021 (3)	0.027 (3)	0.001 (3)	−0.008 (3)	0.001 (3)
C7	0.016 (3)	0.020 (3)	0.022 (3)	0.001 (2)	0.005 (2)	−0.001 (2)
C315	0.032 (4)	0.032 (4)	0.034 (4)	−0.010 (3)	−0.016 (3)	0.020 (3)
C235	0.025 (3)	0.017 (3)	0.024 (3)	−0.001 (2)	0.000 (3)	0.001 (2)
C233	0.021 (3)	0.024 (3)	0.034 (4)	−0.006 (3)	0.013 (3)	−0.001 (3)
C133	0.026 (4)	0.035 (4)	0.045 (4)	−0.007 (3)	−0.007 (3)	0.012 (3)
C125	0.018 (3)	0.026 (3)	0.025 (3)	0.001 (2)	0.000 (2)	−0.004 (3)
C216	0.012 (3)	0.042 (4)	0.034 (4)	−0.003 (3)	0.004 (3)	−0.016 (3)
C226	0.031 (3)	0.013 (3)	0.021 (3)	−0.005 (2)	0.003 (3)	−0.002 (2)
C324	0.051 (5)	0.017 (3)	0.039 (4)	−0.005 (3)	0.007 (3)	0.003 (3)
C114	0.032 (4)	0.036 (4)	0.024 (3)	−0.015 (3)	0.011 (3)	−0.013 (3)
C214	0.030 (4)	0.044 (4)	0.020 (3)	0.002 (3)	−0.004 (3)	−0.020 (3)
C135	0.022 (3)	0.024 (3)	0.074 (6)	0.004 (3)	0.021 (4)	0.014 (4)
C316	0.022 (3)	0.025 (3)	0.034 (4)	−0.005 (3)	−0.006 (3)	0.002 (3)
C134	0.016 (3)	0.022 (3)	0.085 (6)	−0.001 (3)	−0.003 (4)	0.021 (4)
C3	0.023 (3)	0.024 (3)	0.021 (3)	−0.001 (2)	0.008 (3)	−0.002 (3)
C136	0.022 (3)	0.025 (3)	0.040 (4)	0.003 (3)	0.015 (3)	0.006 (3)
C314	0.029 (4)	0.019 (3)	0.059 (5)	−0.002 (3)	−0.014 (3)	0.008 (3)
C323	0.031 (4)	0.030 (3)	0.028 (4)	−0.017 (3)	−0.004 (3)	−0.003 (3)
C232	0.017 (3)	0.022 (3)	0.029 (3)	0.001 (2)	0.006 (2)	0.001 (3)

### Geometric parameters (Å, °)

**Table:**

Ag—O1	2.630 (4)	C311—C312	1.393 (8)
Ag—O2	2.597 (4)	C4—C5	1.380 (8)
Ag—P1	2.5412 (14)	C4—C3	1.391 (8)
Ag—P2	2.5198 (14)	C4—H4	0.9500
Ag—P3	2.5160 (14)	C322—C323	1.400 (8)
Br7—C7	1.901 (6)	C322—H322	0.9500
Br5—C5	1.915 (5)	C131—C136	1.391 (8)
Br3—C3	1.906 (6)	C222—H222	0.9500
P2—C221	1.838 (6)	C336—H336	0.9500
P2—C211	1.838 (5)	C5—C6	1.386 (8)
P2—C231	1.849 (5)	C334—H334	0.9500
P1—C131	1.830 (5)	C124—C123	1.389 (8)
P1—C121	1.833 (5)	C124—C125	1.390 (8)
P1—C111	1.837 (6)	C124—H124	0.9500
P3—C321	1.823 (6)	C236—C235	1.382 (8)
P3—C331	1.829 (6)	C236—C231	1.398 (7)
P3—C311	1.833 (6)	C236—H236	0.9500
C1—O1	1.252 (6)	C215—C214	1.368 (9)
C1—C7	1.429 (8)	C215—C216	1.387 (8)
C1—C2	1.502 (8)	C215—H215	0.9500
O2—C2	1.238 (7)	C325—C324	1.387 (9)
C331—C336	1.385 (8)	C325—C326	1.399 (8)
C331—C332	1.413 (7)	C325—H325	0.9500
C335—C336	1.380 (8)	C6—C7	1.382 (8)
C335—C334	1.398 (8)	C6—H6	0.9500
C335—H335	0.9500	C112—H112	0.9500
C221—C226	1.396 (8)	C123—H123	0.9500
C221—C222	1.402 (8)	C213—C214	1.367 (8)
C121—C126	1.385 (7)	C213—H213	0.9500
C121—C122	1.400 (7)	C231—C232	1.393 (7)
C111—C116	1.394 (8)	C313—C314	1.356 (9)
C111—C112	1.401 (8)	C313—C312	1.385 (8)
C211—C212	1.388 (8)	C313—H313	0.9500
C211—C216	1.395 (7)	C126—C125	1.385 (8)
C132—C133	1.375 (8)	C126—H126	0.9500
C132—C131	1.402 (8)	C312—H312	0.9500
C132—H132	0.9500	C115—C114	1.379 (8)
C234—C233	1.382 (8)	C115—H115	0.9500
C234—C235	1.388 (8)	C326—H326	0.9500
C234—H234	0.9500	C315—C314	1.376 (9)
C333—C334	1.377 (8)	C315—C316	1.393 (8)
C333—C332	1.379 (8)	C315—H315	0.9500
C333—H333	0.9500	C235—H235	0.9500
C223—C224	1.376 (8)	C233—C232	1.386 (8)
C223—C222	1.378 (8)	C233—H233	0.9500
C223—H223	0.9500	C133—C134	1.370 (10)
C116—C115	1.387 (8)	C133—H133	0.9500
C116—H116	0.9500	C125—H125	0.9500
C2—C3	1.443 (8)	C216—H216	0.9500
C225—C226	1.379 (8)	C226—H226	0.9500
C225—C224	1.384 (9)	C324—C323	1.375 (9)
C225—H225	0.9500	C324—H324	0.9500
C332—H332	0.9500	C114—H114	0.9500
C122—C123	1.377 (8)	C214—H214	0.9500
C122—H122	0.9500	C135—C134	1.392 (11)
C224—H224	0.9500	C135—C136	1.396 (8)
C321—C322	1.395 (8)	C135—H135	0.9500
C321—C326	1.398 (8)	C316—H316	0.9500
C113—C112	1.372 (8)	C134—H134	0.9500
C113—C114	1.380 (9)	C136—H136	0.9500
C113—H113	0.9500	C314—H314	0.9500
C212—C213	1.377 (8)	C323—H323	0.9500
C212—H212	0.9500	C232—H232	0.9500
C311—C316	1.392 (8)		
			
O2—Ag—O1	59.36 (12)	C331—C336—H336	119.7
P3—Ag—P2	112.98 (5)	C4—C5—C6	129.8 (5)
P3—Ag—P1	112.59 (5)	C4—C5—Br5	115.4 (4)
P2—Ag—P1	118.42 (5)	C6—C5—Br5	114.7 (4)
P3—Ag—O2	105.72 (10)	C333—C334—C335	119.5 (6)
P2—Ag—O2	126.43 (10)	C333—C334—H334	120.3
P1—Ag—O2	75.45 (9)	C335—C334—H334	120.3
P3—Ag—O1	81.79 (9)	C123—C124—C125	120.0 (5)
P2—Ag—O1	90.69 (9)	C123—C124—H124	120.0
P1—Ag—O1	134.81 (9)	C125—C124—H124	120.0
C221—P2—C211	105.1 (2)	C235—C236—C231	119.5 (5)
C221—P2—C231	102.8 (2)	C235—C236—H236	120.3
C211—P2—C231	101.1 (2)	C231—C236—H236	120.3
C221—P2—Ag	108.77 (17)	C214—C215—C216	119.4 (6)
C211—P2—Ag	114.75 (18)	C214—C215—H215	120.3
C231—P2—Ag	122.54 (18)	C216—C215—H215	120.3
C131—P1—C121	102.7 (2)	C324—C325—C326	120.0 (6)
C131—P1—C111	103.7 (3)	C324—C325—H325	120.0
C121—P1—C111	102.3 (2)	C326—C325—H325	120.0
C131—P1—Ag	115.06 (19)	C7—C6—C5	127.6 (5)
C121—P1—Ag	114.32 (18)	C7—C6—H6	116.2
C111—P1—Ag	116.87 (18)	C5—C6—H6	116.2
C321—P3—C331	104.5 (3)	C113—C112—C111	120.1 (6)
C321—P3—C311	105.2 (2)	C113—C112—H112	119.9
C331—P3—C311	103.2 (3)	C111—C112—H112	119.9
C321—P3—Ag	115.31 (18)	C122—C123—C124	119.9 (5)
C331—P3—Ag	113.83 (17)	C122—C123—H123	120.0
C311—P3—Ag	113.60 (19)	C124—C123—H123	120.0
O1—C1—C7	120.4 (5)	C214—C213—C212	119.7 (6)
O1—C1—C2	115.2 (5)	C214—C213—H213	120.1
C7—C1—C2	124.4 (5)	C212—C213—H213	120.1
C1—O1—Ag	123.6 (4)	C232—C231—C236	119.6 (5)
C2—O2—Ag	124.2 (4)	C232—C231—P2	124.4 (4)
C336—C331—C332	118.6 (5)	C236—C231—P2	115.9 (4)
C336—C331—P3	125.6 (4)	C314—C313—C312	121.5 (7)
C332—C331—P3	115.7 (4)	C314—C313—H313	119.2
C336—C335—C334	120.5 (6)	C312—C313—H313	119.2
C336—C335—H335	119.8	C125—C126—C121	120.8 (5)
C334—C335—H335	119.8	C125—C126—H126	119.6
C226—C221—C222	119.2 (5)	C121—C126—H126	119.6
C226—C221—P2	119.2 (4)	C313—C312—C311	119.5 (6)
C222—C221—P2	121.4 (4)	C313—C312—H312	120.2
C126—C121—C122	119.0 (5)	C311—C312—H312	120.2
C126—C121—P1	118.3 (4)	C114—C115—C116	120.5 (6)
C122—C121—P1	122.6 (4)	C114—C115—H115	119.7
C116—C111—C112	118.7 (5)	C116—C115—H115	119.7
C116—C111—P1	122.5 (4)	C321—C326—C325	120.1 (6)
C112—C111—P1	118.7 (4)	C321—C326—H326	120.0
C212—C211—C216	119.4 (5)	C325—C326—H326	120.0
C212—C211—P2	118.8 (4)	C6—C7—C1	132.6 (5)
C216—C211—P2	121.8 (4)	C6—C7—Br7	113.4 (4)
C133—C132—C131	120.9 (6)	C1—C7—Br7	113.9 (4)
C133—C132—H132	119.6	C314—C315—C316	120.7 (6)
C131—C132—H132	119.6	C314—C315—H315	119.6
C233—C234—C235	119.7 (5)	C316—C315—H315	119.6
C233—C234—H234	120.2	C236—C235—C234	120.8 (5)
C235—C234—H234	120.2	C236—C235—H235	119.6
C334—C333—C332	120.4 (6)	C234—C235—H235	119.6
C334—C333—H333	119.8	C234—C233—C232	120.2 (6)
C332—C333—H333	119.8	C234—C233—H233	119.9
C224—C223—C222	120.1 (6)	C232—C233—H233	119.9
C224—C223—H223	120.0	C134—C133—C132	120.0 (7)
C222—C223—H223	120.0	C134—C133—H133	120.0
C115—C116—C111	120.2 (5)	C132—C133—H133	120.0
C115—C116—H116	119.9	C126—C125—C124	119.7 (5)
C111—C116—H116	119.9	C126—C125—H125	120.1
O2—C2—C3	120.6 (5)	C124—C125—H125	120.1
O2—C2—C1	116.4 (5)	C215—C216—C211	119.8 (6)
C3—C2—C1	122.7 (5)	C215—C216—H216	120.1
C226—C225—C224	120.1 (6)	C211—C216—H216	120.1
C226—C225—H225	120.0	C225—C226—C221	120.0 (6)
C224—C225—H225	120.0	C225—C226—H226	120.0
C333—C332—C331	120.5 (5)	C221—C226—H226	120.0
C333—C332—H332	119.8	C323—C324—C325	119.9 (6)
C331—C332—H332	119.8	C323—C324—H324	120.0
C123—C122—C121	120.6 (5)	C325—C324—H324	120.0
C123—C122—H122	119.7	C115—C114—C113	119.3 (6)
C121—C122—H122	119.7	C115—C114—H114	120.4
C223—C224—C225	120.5 (6)	C113—C114—H114	120.4
C223—C224—H224	119.8	C213—C214—C215	121.5 (6)
C225—C224—H224	119.8	C213—C214—H214	119.2
C322—C321—C326	119.7 (5)	C215—C214—H214	119.2
C322—C321—P3	122.6 (4)	C134—C135—C136	119.3 (7)
C326—C321—P3	117.5 (4)	C134—C135—H135	120.4
C112—C113—C114	121.1 (6)	C136—C135—H135	120.4
C112—C113—H113	119.4	C311—C316—C315	119.5 (6)
C114—C113—H113	119.4	C311—C316—H316	120.3
C213—C212—C211	120.1 (5)	C315—C316—H316	120.3
C213—C212—H212	119.9	C133—C134—C135	120.8 (6)
C211—C212—H212	119.9	C133—C134—H134	119.6
C316—C311—C312	119.2 (5)	C135—C134—H134	119.6
C316—C311—P3	123.0 (5)	C4—C3—C2	132.3 (6)
C312—C311—P3	117.7 (4)	C4—C3—Br3	114.8 (4)
C5—C4—C3	127.2 (6)	C2—C3—Br3	112.9 (4)
C5—C4—H4	116.4	C131—C136—C135	120.3 (7)
C3—C4—H4	116.4	C131—C136—H136	119.8
C321—C322—C323	119.3 (5)	C135—C136—H136	119.8
C321—C322—H322	120.4	C313—C314—C315	119.5 (6)
C323—C322—H322	120.4	C313—C314—H314	120.3
C136—C131—C132	118.7 (5)	C315—C314—H314	120.3
C136—C131—P1	123.8 (5)	C324—C323—C322	121.1 (6)
C132—C131—P1	117.5 (4)	C324—C323—H323	119.4
C223—C222—C221	120.1 (6)	C322—C323—H323	119.4
C223—C222—H222	120.0	C233—C232—C231	120.2 (5)
C221—C222—H222	120.0	C233—C232—H232	119.9
C335—C336—C331	120.5 (5)	C231—C232—H232	119.9
C335—C336—H336	119.7		
			
P3—Ag—P2—C221	67.3 (2)	C216—C211—C212—C213	0.9 (9)
P1—Ag—P2—C221	−67.4 (2)	P2—C211—C212—C213	179.0 (5)
O2—Ag—P2—C221	−159.9 (2)	C321—P3—C311—C316	94.6 (5)
O1—Ag—P2—C221	148.7 (2)	C331—P3—C311—C316	−14.6 (5)
P3—Ag—P2—C211	−175.37 (19)	Ag—P3—C311—C316	−138.4 (4)
P1—Ag—P2—C211	49.9 (2)	C321—P3—C311—C312	−89.3 (5)
O2—Ag—P2—C211	−42.6 (2)	C331—P3—C311—C312	161.5 (4)
O1—Ag—P2—C211	−94.0 (2)	Ag—P3—C311—C312	37.7 (5)
P3—Ag—P2—C231	−52.3 (2)	C326—C321—C322—C323	0.2 (9)
P1—Ag—P2—C231	173.0 (2)	P3—C321—C322—C323	173.8 (5)
O2—Ag—P2—C231	80.5 (2)	C133—C132—C131—C136	−2.7 (9)
O1—Ag—P2—C231	29.1 (2)	C133—C132—C131—P1	177.6 (5)
P3—Ag—P1—C131	49.6 (2)	C121—P1—C131—C136	−101.7 (5)
P2—Ag—P1—C131	−175.5 (2)	C111—P1—C131—C136	4.6 (5)
O2—Ag—P1—C131	−51.7 (2)	Ag—P1—C131—C136	133.4 (4)
O1—Ag—P1—C131	−51.7 (2)	C121—P1—C131—C132	78.0 (5)
P3—Ag—P1—C121	−68.98 (19)	C111—P1—C131—C132	−175.7 (4)
P2—Ag—P1—C121	65.92 (19)	Ag—P1—C131—C132	−46.8 (5)
O2—Ag—P1—C121	−170.2 (2)	C224—C223—C222—C221	−0.4 (8)
O1—Ag—P1—C121	−170.2 (2)	C226—C221—C222—C223	−2.1 (8)
P3—Ag—P1—C111	171.6 (2)	P2—C221—C222—C223	−177.3 (4)
P2—Ag—P1—C111	−53.5 (2)	C334—C335—C336—C331	−1.4 (8)
O2—Ag—P1—C111	70.3 (2)	C332—C331—C336—C335	2.4 (8)
O1—Ag—P1—C111	70.3 (2)	P3—C331—C336—C335	179.1 (4)
P2—Ag—P3—C321	−110.3 (2)	C3—C4—C5—C6	−6.4 (11)
P1—Ag—P3—C321	27.1 (2)	C3—C4—C5—Br5	176.8 (5)
O2—Ag—P3—C321	107.6 (2)	C332—C333—C334—C335	1.3 (9)
O1—Ag—P3—C321	162.4 (2)	C336—C335—C334—C333	−0.4 (9)
P2—Ag—P3—C331	10.4 (2)	C4—C5—C6—C7	2.4 (11)
P1—Ag—P3—C331	147.86 (19)	Br5—C5—C6—C7	179.3 (5)
O2—Ag—P3—C331	−131.7 (2)	C114—C113—C112—C111	−1.5 (9)
O1—Ag—P3—C331	−76.8 (2)	C116—C111—C112—C113	1.3 (8)
P2—Ag—P3—C311	128.2 (2)	P1—C111—C112—C113	176.8 (4)
P1—Ag—P3—C311	−94.4 (2)	C121—C122—C123—C124	0.9 (9)
O2—Ag—P3—C311	−13.9 (2)	C125—C124—C123—C122	0.3 (9)
O1—Ag—P3—C311	41.0 (2)	C211—C212—C213—C214	−1.2 (9)
C7—C1—O1—Ag	177.8 (4)	C235—C236—C231—C232	0.8 (8)
C2—C1—O1—Ag	−3.2 (6)	C235—C236—C231—P2	−175.8 (4)
P3—Ag—O1—C1	−107.4 (4)	C221—P2—C231—C232	16.0 (5)
P2—Ag—O1—C1	139.5 (4)	C211—P2—C231—C232	−92.4 (5)
P1—Ag—O1—C1	6.4 (5)	Ag—P2—C231—C232	138.4 (4)
O2—Ag—O1—C1	6.4 (4)	C221—P2—C231—C236	−167.5 (4)
P3—Ag—O2—C2	59.9 (4)	C211—P2—C231—C236	84.0 (4)
P2—Ag—O2—C2	−75.5 (5)	Ag—P2—C231—C236	−45.1 (5)
P1—Ag—O2—C2	169.7 (5)	C122—C121—C126—C125	1.3 (8)
O1—Ag—O2—C2	−10.3 (4)	P1—C121—C126—C125	−175.3 (4)
C321—P3—C331—C336	−0.2 (5)	C314—C313—C312—C311	1.2 (9)
C311—P3—C331—C336	109.6 (5)	C316—C311—C312—C313	−0.1 (8)
Ag—P3—C331—C336	−126.8 (4)	P3—C311—C312—C313	−176.4 (4)
C321—P3—C331—C332	176.6 (4)	C111—C116—C115—C114	0.0 (9)
C311—P3—C331—C332	−73.6 (4)	C322—C321—C326—C325	−0.8 (9)
Ag—P3—C331—C332	50.0 (4)	P3—C321—C326—C325	−174.7 (5)
C211—P2—C221—C226	164.5 (4)	C324—C325—C326—C321	0.9 (10)
C231—P2—C221—C226	59.0 (5)	C5—C6—C7—C1	10.2 (11)
Ag—P2—C221—C226	−72.2 (4)	C5—C6—C7—Br7	−173.9 (5)
C211—P2—C221—C222	−20.4 (5)	O1—C1—C7—C6	173.6 (6)
C231—P2—C221—C222	−125.8 (4)	C2—C1—C7—C6	−5.3 (10)
Ag—P2—C221—C222	102.9 (4)	O1—C1—C7—Br7	−2.2 (7)
C131—P1—C121—C126	−156.8 (4)	C2—C1—C7—Br7	178.9 (4)
C111—P1—C121—C126	95.9 (5)	C231—C236—C235—C234	−1.3 (8)
Ag—P1—C121—C126	−31.5 (5)	C233—C234—C235—C236	0.3 (9)
C131—P1—C121—C122	26.8 (5)	C235—C234—C233—C232	1.1 (9)
C111—P1—C121—C122	−80.5 (5)	C131—C132—C133—C134	0.5 (9)
Ag—P1—C121—C122	152.1 (4)	C121—C126—C125—C124	−0.2 (8)
C131—P1—C111—C116	−111.3 (5)	C123—C124—C125—C126	−0.6 (9)
C121—P1—C111—C116	−4.7 (5)	C214—C215—C216—C211	0.8 (10)
Ag—P1—C111—C116	121.0 (4)	C212—C211—C216—C215	−0.7 (9)
C131—P1—C111—C112	73.4 (5)	P2—C211—C216—C215	−178.8 (5)
C121—P1—C111—C112	−180.0 (4)	C224—C225—C226—C221	−2.4 (9)
Ag—P1—C111—C112	−54.3 (5)	C222—C221—C226—C225	3.6 (8)
C221—P2—C211—C212	118.0 (4)	P2—C221—C226—C225	178.8 (4)
C231—P2—C211—C212	−135.3 (4)	C326—C325—C324—C323	−0.5 (11)
Ag—P2—C211—C212	−1.4 (5)	C116—C115—C114—C113	−0.3 (9)
C221—P2—C211—C216	−63.9 (5)	C112—C113—C114—C115	1.0 (9)
C231—P2—C211—C216	42.7 (5)	C212—C213—C214—C215	1.2 (10)
Ag—P2—C211—C216	176.7 (4)	C216—C215—C214—C213	−1.0 (11)
C112—C111—C116—C115	−0.5 (8)	C312—C311—C316—C315	−0.6 (8)
P1—C111—C116—C115	−175.8 (4)	P3—C311—C316—C315	175.4 (4)
Ag—O2—C2—C3	−160.9 (4)	C314—C315—C316—C311	0.4 (9)
Ag—O2—C2—C1	12.9 (7)	C132—C133—C134—C135	1.6 (10)
O1—C1—C2—O2	−6.1 (7)	C136—C135—C134—C133	−1.6 (9)
C7—C1—C2—O2	172.9 (5)	C5—C4—C3—C2	−10.0 (11)
O1—C1—C2—C3	167.6 (5)	C5—C4—C3—Br3	173.3 (5)
C7—C1—C2—C3	−13.4 (8)	O2—C2—C3—C4	−163.7 (6)
C334—C333—C332—C331	−0.3 (8)	C1—C2—C3—C4	22.9 (10)
C336—C331—C332—C333	−1.5 (8)	O2—C2—C3—Br3	13.0 (7)
P3—C331—C332—C333	−178.6 (4)	C1—C2—C3—Br3	−160.4 (4)
C126—C121—C122—C123	−1.6 (8)	C132—C131—C136—C135	2.7 (9)
P1—C121—C122—C123	174.8 (4)	P1—C131—C136—C135	−177.5 (4)
C222—C223—C224—C225	1.6 (9)	C134—C135—C136—C131	−0.6 (9)
C226—C225—C224—C223	−0.2 (9)	C312—C313—C314—C315	−1.5 (9)
C331—P3—C321—C322	110.9 (5)	C316—C315—C314—C313	0.6 (9)
C311—P3—C321—C322	2.6 (6)	C325—C324—C323—C322	−0.1 (10)
Ag—P3—C321—C322	−123.3 (4)	C321—C322—C323—C324	0.3 (9)
C331—P3—C321—C326	−75.3 (5)	C234—C233—C232—C231	−1.5 (9)
C311—P3—C321—C326	176.4 (5)	C236—C231—C232—C233	0.5 (9)
Ag—P3—C321—C326	50.4 (5)	P2—C231—C232—C233	176.9 (4)
